# A regulatory effect of IL-21 on T follicular helper-like cell and B cell in rheumatoid arthritis

**DOI:** 10.1186/ar4100

**Published:** 2012-11-23

**Authors:** Rui Liu, Qian Wu, Dinglei Su, Nan Che, Haifeng Chen, Linyu Geng, Jinyun Chen, Wanjun Chen, Xia Li, Lingyun Sun

**Affiliations:** 1 Department of Immunology and Rheumatology, Drum Tower Clinical Medical College of Nanjing Medical University, Nanjing, Jiangsu, 210008, PR China; 2 Department of Immunology and Rheumatology, The Affiliated Drum Tower Hospital of Nanjing University Medical School, Nanjing, Jiangsu, 210008, PR China; 3 Mucosal Immunology Section, National Institute of Dental and Craniofacial Research, National Institutes of Health, Bethesda, Maryland, USA; 4 Department of Biochemistry, Dalian Medical University, Dalian, Liaoning, 116044, PR China

## Abstract

**Introduction:**

Interleukin (IL)-21 is a member of type I cytokine family. Recent studies indicate that IL-21 can promote T follicular helper (Tfh) cell differentiation and survival, a specialized T cell subset which provides help for B cell. It can also regulate the activation, proliferation and differentiation of human B cell and immunoglobulin (Ig) production as well as isotype switching of plasma cell. Rheumatoid arthritis (RA) is characterized by auto-antibodies overproduction such as rheumatoid factor (RF) and anti-cyclic citrullinated peptide (anti-CCP) antibody, suggesting a pivotal role of Tfh cell and B cell in the pathogenesis of RA. This study aimed to investigate whether IL-21 had a regulatory effect on Tfh cell and B cell in RA.

**Methods:**

Serum IL-21 concentrations were measured by ELISA. The correlations between serum IL-21 levels and clinical features of RA patients were analyzed by Spearman's rank test. The percentages of Tfh-like cells, IL-21 receptor (R) expression on Tfh-like cells and B cells in peripheral blood (PB) were analyzed by flow cytometry. Peripheral blood mononuclear cells (PBMC) were stimulated by rIL-21 (100 ng/ml) in the presence or absence of anti-CD40 and/or anti-IgM, and changes of IL-21R, activation-associated surface markers (CD25, CD69 and CD40), the proliferation, apoptosis and differentiation of B cells were analyzed by flow cytometry. Production of IgG and IgM in the culture supernatants was determined by ELISA.

**Results:**

The results showed that the serum IL-21 levels in RA patients were significantly higher than that of healthy controls (HC). IL-21 concentrations were positively correlated with 28-joint count disease activity score (DAS28) and anti-CCP antibody in RA patients with high IL-21 levels. Furthermore, the frequencies of peripheral CXCR5^+^PD-1^+^CD4^+ ^Tfh-like cells markedly increased in RA patients and the percentages of Tfh-like cells were positively correlated with DAS28 and anti-CCP antibody levels. Moreover, elevated IL-21 levels were also correlated with the frequencies of Tfh-like cells. IL-21R expression on both Tfh-like cells and B cells were significantly enhanced in RA patients. In cultures vitro, exogenous IL-21 upregulated IL-21R expression and activation-associated surface markers on B cells and promoted more B cell proliferation in RA than in HC. This IL-21-mediated effect could be reversed by IL-21R-specific neutralizing antibody. Importantly, IL-21 promoted more differentiation of B cell into plasmablast and higher levels of IgG and IgM production in RA than in HC.

**Conclusions:**

Increased serum IL-21 levels in RA patients correlate with DAS28, anti-CCP antibody and frequencies of Tfh-like cells. IL-21 supports B cell activation, proliferation and antibody secretion via IL-21R pathway. Thus, IL-21 may be involved in the pathogenesis of RA and antagonizing IL-21 could be a novel strategy for the therapy of RA.

## Introduction

Interleukin (IL)-21 is a member of the type I cytokine family and can be secreted by CD4^+ ^T cells including T follicular helper (Tfh) cells, Th17 cells and natural killer (NK) T cells [1]. IL-21 signals through the common cytokine receptor γ chain in combination with its functional receptor, IL-21 receptor (R) which is mainly expressed on B cells and also on T cells, NK cells, dendritic cells, epithelial cells and fibroblasts [2-4].

It has been reported that IL-21 is able to enhance the proliferation and effector characteristics of activated CD4^+ ^and CD8^+ ^T cells [5] and limit the differentiation of inducible regulatory T cells [6-8]. IL-21 can also modulate Tfh cell differentiation via the upregulation of Bcl-6, the transcription factor of Tfh cells [9]. The Tfh cell is a specialized T cell subset, which is characterized by increased expression of molecules, including CXCR5, PD-1, ICOS, CD40L and IL-21 and decreased expression of CCR7 [10]. Expressing these molecules allows Tfh cell migration into the germinal center (GC) to provide help for B cell growth, differentiation and class switching [11-13]. Reportedly, exposure of CD4^+ ^T cells to IL-21 drives them to differentiate into a Tfh cell subset partly through modulation of the expression of CXCR5 and CCR7 by IL-21 in an autocrine manner [14,15]. Also, Tfh cell regulation of B cell proliferation, differentiation and antibody production is via the secretion of IL-21 [16-18].

Moreover, IL-21 can directly act on B cells. IL-21 co-stimulation is capable of promoting plasma cells differentiation from CD27^+ ^memory B cells, inducing class switch recombination and stimulating poorly responsive naive cord blood B cells into IgG-secreting plasma cells in humans [11]. In addition, antigen-specific memory B cells and plasma cells fail to expand and IgG production is significantly impaired following secondary immunization of IL-21R.KO mice [19]. Furthermore, IL-21 acts in a B cell-intrinsic fashion to control GC formation [9]. The absence of IL-21 signaling profoundly affects GC persistence and function, influencing its proliferation, transition into memory B cells, and affinity maturation [20]. Thus, the effect of IL-21 on B cells may contribute to the development of autoimmune diseases.

Rheumatoid arthritis (RA) is characterized by persistent synovitis and systemic inflammation, frequently leading to cartilage and bone destruction. Although the etiology and pathology remain elusive, auto-antibodies to citrullinated cyclic peptides (CCP) and rheumatoid factor (RF) have been indicated to be associated with the disease course [21-25]. When transferring auto-antibodies to mice with certain genetic backgrounds, they may provoke articular inflammation [26,27]. Importantly, B cells are the primary source of RF and anti-CCP auto-antibodies and Tfh cells assist B cells, suggesting a critical role of Tfh and B cells in the pathogenesis of RA. Recent studies observe that IL-21R transcript is expressed by synovial macrophages and fibroblasts from RA patients but not from patients with osteoarthritis (OA) [28] and IL-21R deficiency in the K/BxN mouse model of inflammatory arthritis is sufficient to protect it from arthritis [29]. However, the effect of IL-21 regulation on Tfh and B cells in the pathogenesis of RA remains largely unknown.

In this report, we investigated the function of IL-21 on Tfh-like cells and B cells in RA. Our data showed that increased serum IL-21 levels in RA patients correlated positively with 28-joint count disease activity score (DAS28), serum anti-CCP antibodies and frequencies of Tfh-like cells. IL-21 supported B cell activation, proliferation and antibody secretion. Strikingly, blockade of IL-21R markedly inhibited the impact of IL-21 on B cells in RA patients.

## Materials and methods

### Patients and controls

Serum samples were collected from active RA patients (*n *= 120) admitted to the ward of The Affiliated Drum Tower Hospital of Nanjing Medical University, from July 2010 to September 2012. All patients fulfilled the American College of Rheumatology criteria for the classification of RA and they had no other autoimmune or systemic diseases. None of these patients was pregnant or menopausal at the time of the study. Age- and sex-matched healthy controls (HC, *n *= 80) were obtained from the medical examination center. Serum samples were stored at -80°C until used. The study protocol was approved by the ethics committee of The Affiliated Drum Tower Hospital of Nanjing Medical University. Written informed consents were obtained from all patients and controls. Detailed clinical characteristics and laboratory features are shown in Table 1.

**Table 1:** Clinical and laboratory features in 120 patients with rheumatoid arthritis (RA)

Characteristics	Values
Age, yrs	58.95 ± 1.12
Men/women	33/87
Disease duration, yrs	18.02 ± 8.98
DAS28	4.89 ± 0.09
ESR, mm/h	60.86 ± 2.60
CRP, mg/l	38.71 ± 4.03
RF-IgM-positive	89 (74.17)
RF-IgG-positive	66 (55.00)
RF-IgA-positive	72 (60.00)
Anti-CCP-positive	65 (54.17)
Treatment	
DMARDs	9 (7.50)
CS	22 (18.33)
CS+DMARDs	41 (34.17)
Others	48 (40.00)

Clinical characteristics are presented as mean ± SEM or number (%). DAS28, 28-joint count disease activity score; ESR, erythrocyte sedimentation rate; CRP, C-reactive protein; RF, rheumatoid factor; Anti-CCP, anti-cyclic citrullinated peptide antibody; CS, corticosteroid; DMARDs, disease-modifying antirheumatic drugs; Others, non-steroidal anti-inflammatory drugs, traditional Chinese medicine or never taking any medication.

### Enzyme-linked immunosorbent assay (ELISA) for serum IL-21 levels

Serum IL-21 levels in RA patients and HC were measured by human IL-21 ELISA kits (Biolegend, San Diego, CA, USA) according to the instructions of the manufacturer. The plate was read at 450 nm and sensitivity of the ELISA kits used in the experiment was 16 pg/ml.

### Clinical data and inflammation index analysis

All patients were followed up to obtain clinical data on age, sex, disease duration, number of swollen and tender joints, erythrocyte sedimentation rate (ESR), C-reactive protein (CRP), anti-CCP antibodies, RF-IgM, RF-IgG and RF-IgA.

ESR was evaluated by the Westergren method. Values ≤ 15 mm/h for men and 20 mm/h for women were considered normal. CRP was examined by the immunonephelometry method and a value > 8 mg/l was considered positive. Anti-CCP antibody, RF-IgM, RF-IgG and RF-IgA were tested by ELISA, with normal ranges of 0 to 5 RU/ml for RF-IgM, and 0 to 20 RU/ml for RF-IgG and RF-IgA. The DAS28 was calculated as previously described [30].

### Detection of IL-21R and activation marker expression by flow cytometry

Peripheral blood mononuclear cells (PBMC) were isolated from active RA patients or HC using Ficoll density-gradient centrifugation. The cell suspension was washed three times in phosphate- buffered saline (PBS) and then labeled with the following monoclonal antibodies: phycoerythrin (PE)-conjugated anti-IL21R (R&D Systems, Minneapolis, MN, USA), fluorescein isothiocyanate (FITC)- or allophycocyanin (APC)-conjugated anti-CD19, Pecy7-conjugated anti-CD40, PE-conjugated anti-CD25, FITC-conjugated anti-CD69, FITC-conjugated anti-CD4, APC-conjugated anti-CXCR5 and PerCP-Cy5.5-conjugated anti-PD-1 (BD Biosciences, Franklin Lakes, NJ, USA). For surface marker staining, cells were maintained in the dark at 4°C for 30 minutes and then washed twice in PBS. IL-21R on Tfh-like cells and B cells and activation surface markers expression of B cells were analyzed by flow cytometry.

### B cell stimulation by IL-21

PBMC from RA patients or HC were cultured in RPMI 1640 supplemented with 10% fetal calf serum (FCS) and antibiotics (penicillin 100 IU/ml, streptomycin 100 μg/ml; Invitrogen, Camarillo, CA, USA) in a humidified atmosphere of 5% CO_2 _at 37°C. For the studies of IL-21R and B cell activation, PBMC (1*10^6^/well) were added to 96-well plates directly with or without 100 ng/ml recombinant human IL-21 (Abcam, Cambridge, MA, USA). For proliferation studies, PBMC (1*10^6^/well) were treated with 3 μg/ml anti-CD40 antibody (eBioscience, San Diego, CA, USA), 50 ng/ml rIL-4 (PeproTech Inc, Rocky Hill, NJ, USA) and 100 ng/ml rIL-21 (Abcam). For apoptosis studies, PBMC (1*10^6^/well) were stimulated with 3 μg/ml anti-CD40 antibody (eBioscience), and 100 ng/ml rIL-21 (Abcam). For differentiation to plasmablast studies, PBMC (1*10^6^/well) were treated with 3 μg/ml anti-CD40 antibody (eBioscience), 5 μg/ml F(ab)2 goat anti-human IgM (Jackson ImmunoResearch, West Grove, PA, USA) and 100 ng/ml rIL-21 (Abcam). To investigate the possible mechanism of IL-21, 10 ug/ml anti-IL-21R antibody (Biolegend) was included in cell cultures.

### Quantification of cytokines in serum

Serum cytokines were analyzed using Quantibody Human TH17 Array 1 (QAH-TH17-1, RayBiotech, Norcross, GA, USA), according to the manufacturer's specification. Each sample was prepared in triplicate. An Axon scanner 4000B with GenePix software was used to collect fluorescence intensities.

### Proliferation and apoptosis assays

PBMC from RA patients and HC were labeled with 5 μM carboxyfluorescein diacetate succinimidyl ester (CFSE) (Invitrogen) in PBS for 10 minutes at 37°C. An excess of ice-cold RPMI 1640 with 10% FCS was added to the cells to quench the reaction and cells were washed extensively. CFSE-labeled cells (1*10^6^/well) were cultured according to above-mentioned methods. Following 4 days of culture, cells were collected and then stained with APC-conjugated anti-CD19 (BD Biosciences). B cell proliferation was determined by flow cytometry analysis of CFSE fluorescence intensity. To detect apoptotic cells, cultured PBMC were collected and resuspended in 100 μl of 1 × binding buffer (10 mM HEPES (pH 7.4), 140 mM NaCl and 2.5 mM CaCl_2_) and stained with 5 μl of FITC-conjugated Annexin V (BD PharMingen, San Diego, CA, USA) for 15 minutes at room temperature in the dark and then analyzed by flow cytometry.

### Determination of immunoglobin (Ig) levels

After 4 days' culture, secreted IgG and IgM in the culture supernatants were quantitated by ELISA (MABTECH, Nacka Strand, Sweden) according to the instructions of the manufacturer. PE-conjugated anti-CD138 (BD Biosciences) expression was analyzed in cultured cells using flow cytometry.

### Statistical analysis

Data were summarized as means ± standard error of the mean (SEM). Statistical significance was performed by Student's *t*-test and the correlation coefficient between serum IL-21 levels and clinical features in RA patients were analyzed by Spearman's rank test. All statistical analyses were performed using GraphPad Prism software (Graph-Pad, San Diego, CA, USA). A *P*-value < 0.05 was considered significantly different.

## Results

### Positive correlation of increased IL-21 with disease activity and auto-antibody production in RA patients

**Figure 1:**
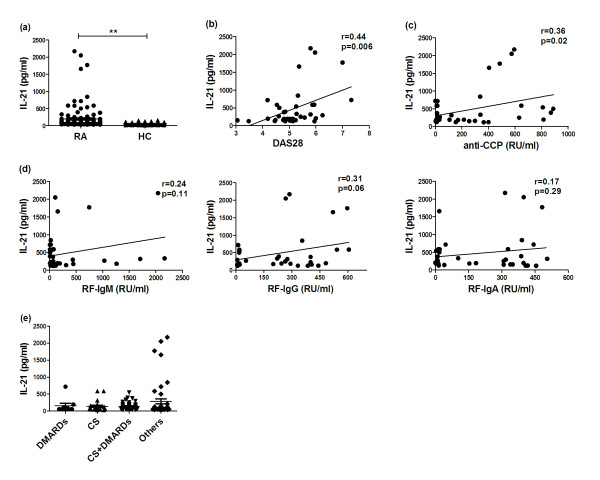
**Positive correlation of high serum IL-21 levels with disease activity and auto-antibody production in patients with rheumatoid arthritis (RA)**. (**a**) Serum IL-21 levels of RA patients (*n *= 120) and healthy controls (HC) (*n *= 80) were measured by ELISA. **(b**) There was positive correlation between serum IL-21 and 28-joint count disease activity score (DAS28) in RA patients with high IL-21 (*n *= 38). (**c**) There was a positive correlation between serum IL-21 and levels of anti-cyclic citrullinated peptide (anti-CCP) antibody in RA patients with high IL-21 (*n *= 38). (**d**) Serum IL-21 had no relationship with levels of rheumatoid factor (RF)-IgM, RF-IgG or RF-IgA in RA patients with high IL-21 (*n *= 38). (**e**) Serum IL-21 levels in RA patients treated with different drugs. **P < 0.01; *P < 0.05. DMARDS, disease-modifying antirheumatic drugs; CS, corticosteroids.

We measured the serum IL-21 levels in RA patients (*n *= 120) and age-matched HC (*n *= 80) by ELISA and revealed that RA patients had significantly higher IL-21 levels than HC subjects (197.60 ± 32.57 vs. 59.10 ± 3.45 pg/ml, *P *< 0.01; Figure 1a).

Then the correlations of IL-21 concentrations with clinical activity and auto-antibody levels were analyzed in the high IL-21 group. IL-21 concentrations > 120.74 pg/ml (mean + 2SD of the HC group) were defined as high IL-21. We found that RA patients with elevated IL-21 levels had a higher DAS28 (*r *= 0.44, *P *= 0.006; Figure 1b). In addition, high IL-21 levels were positively correlated with anti-CCP antibody levels (*r *= 0.36, *P *= 0.02; Figure 1c), but not with the levels of RF-IgM (*r *= 0.24, *P *= 0.11), RF-IgG (*r *= 0.31, *P *= 0.06) and RF-IgA (*r *= 0.17, *P *= 0.29; Figure 1d).

As IL-21 was related to disease activity, we assessed whether drug treatment had any influence on the concentrations of IL-21. RA patients were divided into four groups: 1) patients taking disease modifying anti-rheumatic drugs (DMARDs) including a single DMARD and combination DMARDs; 2) patients taking corticosteroid (CS) including a single CS and CS plus non-DMARDs; 3) patients taking CS plus DMARDs, and 4) patients taking other treatments, including non-steroidal anti-inflammatory drugs (NSAIDs), traditional Chinese medicine, and patients never taking any medication. As expected, IL-21 levels were lowest in patients treated with CS plus DMARDs (133.50 ± 18.22 pg/ml; Figure 1e) and the highest levels of IL-21 were observed in group 4) who were not receiving CS or DMARDs (270.60 ± 71.93 pg/ml; Figure 1e), although this was not statistically significant.

### Positive correlation of increased Tfh-like cells with disease activity and auto-antibody production in RA patients

**Figure 2:**
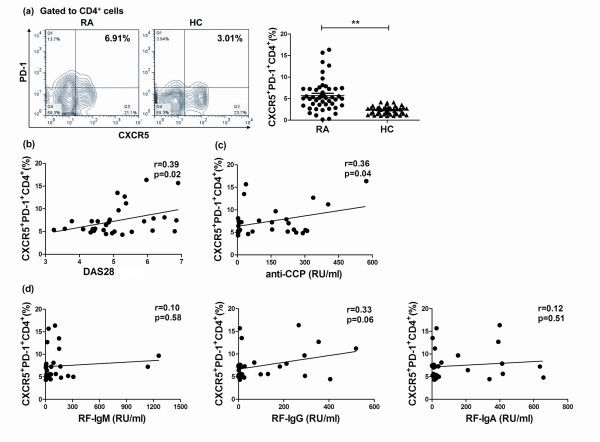
**Positive correlation of increased T follicular helper (Tfh)-like cells with disease activity and auto-antibody production in patients with rheumatoid arthritis (RA)**. (**a**) Frequencies of circulating CXCR5^+^PD-1^+^CD4^+ ^Tfh-like cells were significantly higher in peripheral blood (PB) of RA patients (*n *= 47) than in healthy controls (HC) (*n *= 47). (**b**) There was positive correlation between Tfh-like cells and 28-joint count disease activity score (DAS28) in RA patients with high levels of Tfh-like cells (*n *= 31). (**c**) There was a positive correlation between Tfh-like cells and levels of anti-cyclic citrullinated peptide (anti-CCP antibody) in RA patients with high levels of Tfh-like cells (*n *= 31). (**d**) Tfh-like cells had no relationship with levels of rheumatoid factor (RF)-IgM, RF-IgG and RF-IgA in RA patients with high levels of Tfh-like cells (*n *= 31). **P < 0.01; *P < 0.05.

Recent studies found that Tfh cells could provide help for B cells and allow formation of long-lived antibody responses [10,17,31]. First, the frequencies of circulating CXCR5^+^PD-1^+^CD4^+ ^Tfh-like cells were examined. As shown in Figure 2a, the frequencies of circulating Tfh-like cells were significantly upregulated in peripheral blood (PB) of RA patients (5.71% ± 0.53% vs. 2.32% ± 0.13%, *P *< 0.01; Figure 2a). Serum IL-21 levels in these subjects are shown in Additional file 1a. Furthermore, the correlations of high proportions of Tfh-like cells in RA patients, with clinical activity and auto-antibody production were analyzed. The frequencies of Tfh-like cells > 4.12% (mean + 2SD of the HC group) were defined as high proportions of Tfh-like cells. We found that patients with upregulated frequencies of Tfh-like cells had a higher DAS28 (*r *= 0.39, *P *= 0.02; Figure 2b). In addition, the percentages of Tfh-like cells were positively correlated with anti-CCP antibodies (*r *= 0.36, *P *= 0.04; Figure 2c), but not with RF-IgM (*r *= 0.10, *P *= 0.58), RF-IgG (*r *= 0.33, *P *= 0.06) and RF-IgA (*r *= 0.12, *P *= 0.51; Figure 2d) among the RA patients with high frequency of Tfh-like cells.

### Positive correlation of increased IL-21 with the frequencies of Tfh-like cells in RA patients

**Figure 3:**
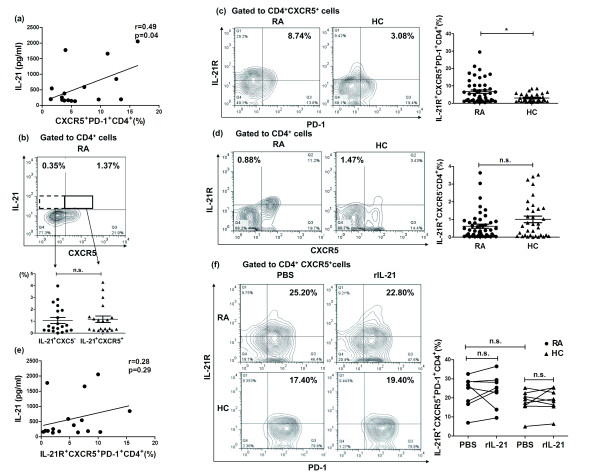
**Positive correlation of increased serum IL-21 with circulating T follicular helper (Tfh)-like cells in patients with rheumatoid arthritis (RA)**. (**a**) Serum IL-21 positively correlated with the percentages of circulating Tfh-like cells in RA patients with high IL-21 (*n *= 16). (**b**) IL-21 derived from both CXCR5^+^CD4^+ ^T cells and CXCR5^-^CD4^+ ^T cells in RA patients (*n *= 20). (**c**) IL-21R expression on Tfh-like cells in peripheral blood (PB) from RA patients (*n *= 47) was substantially upregulated compared to healthy controls (HC) (*n *= 34). (**d**) IL-21R expression on CXCR5^-^CD4^+^T cells was unchanged in RA patients. (**e**) IL-21 had no relationship with IL-21R expression on Tfh-like cells in RA patients with high IL-21 (*n *= 16). (**f**) Peripheral blood mononuclear cells (PBMC) isolated from either RA patients (*n *= 8) or HC (*n *= 8) were cultured with 10 μg/ml phytohemagglutinin (PHA; Sigma) in the presence and absence of rIL-21 (100 ng/ml), and IL-21R expression on Tfh-like cells was analyzed after 48 h.*P < 0.05; n.s., not significant. PBS, phosphate-buffered saline.

Because the source of IL-21 was Tfh cells, Th17 cells and NK T cells, we next investigated the correlation between IL-21 levels and the percentages of Tfh-like cells in the group of RA patients with high IL-21. We found that IL-21 levels positively correlated with the percentages of Tfh-like cells (*r *= 0.49, *P *= 0.04; Figure 3a). Flow cytometry analyses showed that IL-21 was derived from both CXCR5^+^CD4^+ ^T cells and CXCR5^-^CD4^+ ^T cells (1.17% ± 0.27% vs. 1.07% ± 0.25%, *P *> 0.05; Figure 3b).

To investigate a direct function of IL-21 on Tfh-like cells in RA, we examined IL-21R expression on Tfh-like cells. IL-21R expression on Tfh-like cells in RA patients was substantially augmented compared to HC (6.64% ± 0.97% vs. 3.03% ± 0.40%, *P *< 0.05; Figure 3c), but this phenomenon was not detected on CXCR5^-^CD4^+^T cells (0.62% ± 0.11% vs. 1.00% ± 0.19%, *P *> 0.05; Figure 3d). Serum IL-21 levels of these subjects are shown in Additional file 1b. Given that IL-21R expression was significantly increased in PB of RA patients, we observed the functional relevance of IL-21 to IL-21R expression in RA. However, patients with elevated IL-21 levels did not have any relationship with IL-21R expression on Tfh-like cells (*r *= 0.28, *P *= 0.29; Figure 3e). We then cultured PBMC with rIL-21 *in vitro *and IL-21R expression was determined. Figure 3f demonstrated that IL-21R expression on Tfh-like cells remained unchanged both in RA patients (23.05% ± 2.98% vs. 24.16% ± 3.07%, *P *> 0.05; Figure 3f) and in HC (17.36% ± 2.19% vs. 18.59% ± 2.23%, *P *> 0.05; Figure 3f). This is consistent with *in vivo *results. Serum IL-21 levels in these subjects are shown in Additional file 1c.

### The effect of IL-21 on B cell activation in RA patients

**Figure 4:**
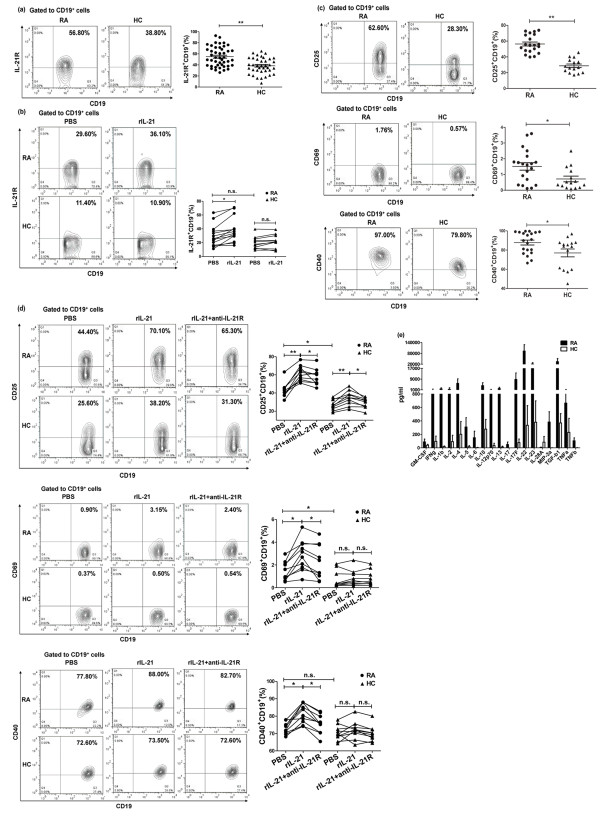
**The effect of IL-21 on IL-21 receptor (R) of B cells and B cell activation in patients with rheumatoid arthritis (RA)**. (**a**) IL-21R expression on B cells was substantially upregulated in RA patients (*n *= 45) compared to healthy controls (HC) (*n *= 38). (**b**) Peripheral blood mononuclear cells (PBMC) from either RA patients (*n *= 14) or HC (*n *= 11) were cultured with or without rIL-21 (100 ng/ml), and IL-21R expression on B cells was analyzed after 48 h. (**c**) Activation marker expressions on B cells were significantly upregulated in RA patients (*n *= 20) compared to HC (*n *= 15). (**d**) PBMC isolated from RA patients (*n *= 10) and HC (*n *= 10) were stimulated with rIL-21 (100 ng/ml) or rIL-21 in combination with anti-IL-21R antibody (10 ug/ml), and activation marker expressions on B cells were examined after 72 h. (**e**) Microarray analysis of cytokines in serum from RA patients (*n *= 6) and HC (*n *= 4). **P < 0.01; *P < 0.05; n.s., not significant. PBS, phosphate-buffered saline.

First, we found that IL-21R expression was significantly upregulated on B cells in RA patients (54.88% ± 2.64% vs. 38.39% ± 2.35%, *P *< 0.01; Figure 4a). Serum IL-21 levels of these subjects are shown in Additional file 2a. Then we cultured PBMC with rIL-21 *in vitro *and IL-21R expression was determined. Figure 4b showed that IL-21R expression on B cells was significantly enhanced in response to rIL-21 in RA patients (29.11% ± 4.11% vs.38.13% ± 4.72%, *P *< 0.05; Figure 4b) but not in HC (19.84% ± 2.68% vs. 21.71% ± 2.95%, *P *> 0.05). Serum IL-21 levels in these subjects were shown in Additional file 2b.

Next, we investigated whether IL-21 was able to regulate B cell activation. We first found that the expressions of B cell activation markers (CD25: 56.34% ± 2.53% vs. 28.78% ± 2.29%, *P *< 0.01; CD69: 1.51% ± 0.24% vs. 0.71% ± 0.18%, *P *< 0.05; CD40: 87.85% ± 2.53% vs. 77.13% ± 4.12%, *P *< 0.05; Figure 4c) were significantly upregulated in RA patients, suggesting B cells are in an activated state in RA. Serum IL-21 levels in these subjects are shown in Additional file 2c. Thus, we supposed that B cell activation was partly due to elevated IL-21 in RA. Then we cultured PBMC in the presence and absence of rIL-21 and the expressions of those markers on B cells were examined. As shown in Figure 4d, inclusion of rIL-21 led to higher expressions of CD25, CD69 and CD40 in RA patients and this response was reversed by anti-IL-21R antibodies (CD25: 43.48% ± 2.63% vs. 61.63% ± 2.63% vs. 57.74% ± 2.89%, *P *< 0.05; CD69: 1.48% ± 0.26% vs. 2.85% ± 0.43% vs. 2.30% ± 0.48%, *P *< 0.05; CD40: 73.14% ± 1.09% vs. 81.77% ± 1.77% vs. 75.22% ± 1.64%, *P *< 0.05; Figure 4d). However, rIL-21 only slightly upregulated CD25 expression on B cells in HC and had no effect on the expression of CD69 and CD40 (CD25: 26.92% ± 1.82% vs. 34.71% ± 2.50% vs. 29.14% ± 1.76%, *P *< 0.05; CD69: 0.73% ± 0.23% vs. 0.80% ± 0.22% vs. 0.79% ± 0.21%, *P *> 0.05; CD40: 70.37% ± 1.30% vs. 71.83% ± 1.63% vs. 70.16% ± 1.42%, *P *> 0.05; Figure 4d). Serum IL-21 levels in these subjects are shown in Additional file 2d. To further explore why IL-21 exposure was prone to activating B cells in RA, we performed microarray analysis of serum from RA patients. We found that several kinds of inflammatory factors were increased (Figure 4e), suggesting an inflammatory microenvironment in RA. Therefore, it indicates that under an inflammatory microenvironment in RA patients, IL-21 is sensitive to activation of B cells.

### The impact of IL-21 on B cell proliferation and differentiation in RA patients

**Figure 5:**
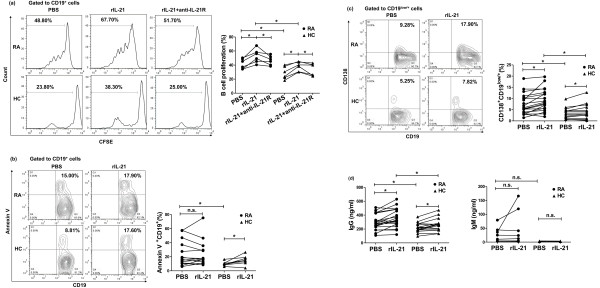
**Impact of IL-21 on B cell proliferation and differentiation in patients with rheumatoid arthritis (RA)**. (**a**) Peripheral blood mononuclear cells (PBMC) isolated from RA patients (*n *= 6) and healthy controls (HC) (*n *= 6) were labeled with carboxyfluorescein diacetate succinimidyl ester (CFSE) and then stimulated with anti-CD40 (3 μg/ml) and rIL-4 (50 ng/ml) in the presence or absence of rIL-21 (100 ng/ml). After 96 h of culture, B cell proliferation was determined. (**b**) PBMC isolated from RA patients (*n *= 14) and HC (*n *= 8) stimulated with anti-CD40 (3 μg/ml) in the presence or absence of rIL-21 (100 ng/ml). Cells stained with Annexin V were examined after 72 h. (**c**) PBMC isolated from RA patients (*n *= 20) and HC (*n *= 15) were stimulated with a combination of anti-CD40 (3 μg/ml) and anti-IgM (5 μg/ml) in the presence or absence of rIL-21 (100 ng/ml). After 96 h, the frequencies of CD138^+^CD19^low/+ ^plasmablasts were determined. **(d**) Secretion of IgG and IgM in culture supernatant was assessed by ELISA *P < 0.05; n.s., not significant. PBS, phosphate-buffered saline.

As previous reports suggested that IL-21 could co-stimulate mature B cells to proliferate in mice [32,33], we studied whether it could promote B cell proliferation in RA patients. Figure 5a demonstrates that in the presence of anti-CD40 and rIL-4, rIL-21 had more effect on B cell proliferation in RA, evidenced by dilution of CFSE. This effect was reversed by anti-IL-21R antibodies in both RA patients (41.65% ± 3.00% vs. 52.82% ± 4.03% vs. 46.50% ± 2.72%, *P *< 0.05; Figure 5a) and HC (28.87% ± 3.66% vs. 38.12% ± 2.66% vs. 33.13% ± 3.59%, *P *< 0.05; Figure 5a). Serum IL-21 levels in these subjects are shown in Additional file 2e. Unexpectedly, IL-21 failed to influence B cell apoptosis in RA patients (24.41% ± 4.82% vs. 24.30% ± 4.89%, *P *> 0.05; Figure 5b), whereas it was able to enhance B cell apoptosis in HC (10.21% ± 1.06% vs. 15.37% ± 2.29%, *P *< 0.05; Figure 5b). These data indicate different effects of IL-21 on B cell apoptosis in RA patients and HC. Serum IL-21 levels in these subjects are shown in Additional file 2f.

As large amounts of RF and anti-CCP antibody appeared in the serum of RA patients, we next observed the effect of IL-21 on the production of IgG and IgM by B cells in RA patients and HC. We showed that rIL-21 upregulated the frequencies of plasmablasts (CD138^+^CD19^low/+^) both in RA patients (7.30% ± 1.01% vs. 9.24% ± 1.08%, *P *< 0.05; Figure 5c) and HC (3.26% ± 0.66% vs. 4.12% ± 0.93%, *P *< 0.05; Figure 5c). However, IL-21-mediated B cell differentiation was stronger in RA patients than in HC. Furthermore, IL-21 induced B cells to produce higher levels of IgG and IgM in RA patients (IgG: 297.20 ± 24.77 vs. 353.70 ± 29.30 ng/ml, *P *< 0.05; IgM: 12.43 ± 4.39 vs. 20.47 ± 9.73 ng/ml, *P *> 0.05; Figure 5d) compared to HC (IgG: 221.60 ± 18.19 vs. 258.50 ± 24.51 ng/ml, *P *< 0.05; IgM: 3.29 ± 0.18 vs. 3.03 ± 0.19 ng/ml, *P *> 0.05; Figure 5d), although there were no marked differences in the secretion of IgM. Serum IL-21 levels in these subjects are shown in Additional file 2g.

## Discussion

Previous studies have detected IL-21R at mRNA and protein levels in synovial tissue samples from RA patients [28,34], supporting the idea that IL-21/IL-21R is implicated in the pathogenesis of RA. In the present study we showed serum IL-21 levels were significantly increased in RA patients. IL-21 concentrations were positively correlated with anti-CCP antibodies in the high IL-21 group of RA patients, suggesting IL-21 might be involved in auto-antibody production. Moreover, the high IL-21 levels were correlated with DAS28, indicating that IL-21 relates to clinical activity, which is consistent with the study by Rasmussen *et al*.[35].

Tfh cells are a subset of T cells that specialize in providing help for B cells. They are characterized by increased expression of molecules including CXCR5, PD-1, ICOS, CD40L and IL-21, and decreased expression of CCR7 [10]. Most of the Tfh cells are located in the light zone of GC in secondary lymphoid tissue in human, but it was reported that human blood CD4^+^CXCR5^+ ^T cells also shared functional properties with Tfh cells [17]. In our study we observed that the frequencies of circulating Tfh-like cells markedly increased in PB from RA patients, suggesting Tfh-like cells might be involved in the pathogenesis of RA. As the function of Tfh cells was assisting B cells to secret antibodies, we next assessed the relationship between Tfh-like cells and anti-CCP antibodies/RF. The results revealed that the high percentages of Tfh-like cells were positively correlated with anti-CCP antibodies but not RF.

Based on the relationship between IL-21 and auto-antibody production, we focused on the role of IL-21 in regulating Tfh-like cells and B cells, two principal cells contributing to antibody secretion. First of all, we found that high IL-21 levels were positively correlated with the frequencies of Tfh-like cells. Furthermore, IL-21R expression on Tfh-like cells was upregulated, confirming that Tfh-like cells are potent responders for IL-21. Reportedly, IL-21 was essential for B cell activation [36] and was the most potent T cell-derived cytokine to induce B cell proliferation from PB, spleen, and tonsil in humans [11]. Our study demonstrated that IL-21 was able to directly promote B cell activation *in vitro *in RA patients. Moreover, IL-21 induced B cell expansion more significantly in RA patients than in HC. A balance between the proliferation and apoptosis of immune cells is needed to maintain homeostasis of the immune system. In this study, IL-21 had different impacts on the apoptosis of B cells in RA patients and HC. In HC, IL-21 promoted B cell apoptosis. A previous study demonstrated that IL-21 stimulation of murine B cells led to a rapid decrease of anti-apoptotic protein Bcl-2 and Bcl-X_L _at mRNA and protein levels [37], which might be a possible mechanism of our result. However, we did not find the influence of IL-21 on B cell apoptosis in RA patients. Notably, our results have shown that IL-21 stimulation promoted B cell activation and expansion in RA. This discrepancy implicates that IL-21 might be prone to inducing B cell activation and expansion instead of apoptosis in RA.

Ig production is a key component in B cell differentiation and the generation of protective humoral immune responses. Accumulated evidence suggests that IL-21 in combination with CD40L and/or anti-IgM is an inducer of plasma cell differentiation [11,38]. Moreover, IL-21 has also been shown to be a critical cytokine that stimulates IgG production compared to IL-2, IL-4 and IL-10 [11,38]. We found that IL-21 induced anti-CD40 and anti-IgM-stimulated B cells to differentiate into plasmablasts, and yielded higher levels of IgG and IgM in RA patients compared to HC, although there were no significant differences in the secretion of IgM. It indicates that the responsibility of IL-21 for Ig production may be stronger in RA patients. Through microarray analysis we found many inflammatory factors levels were upregulated in serum from RA patients, suggesting an inflammatory microenvironment in RA. Among these elevated cytokines, IL-2, IL-4, IL-6, IL-7 and IL-10 are all critical for B cell survival and differentiation [39-43]. We speculate that these cytokines might also play important roles in regulating B cell function in RA, and under this inflammatory microenvironment, IL-21 is prone to inducing B cell activation, expansion and differentiation.

IL-21 has been implicated in autoimmunity disease via the IL-21R pathway. Jang *et al*. addressed the theory that IL-21R deficiency in the K/BxN mouse model of inflammatory arthritis was sufficient to protect it from arthritis [29]. Blocking the IL-21 pathway with IL-21R-Fc fusion protein has been shown to ameliorate the clinical and histologic signs of arthritis and dramatically reduce total IgG1 and inflammatory cytokines levels [44]. Similarly, IL-21R-deficient BXSB.B6-Yaa+/J mice presented none of the abnormal characteristics of systemic lupus erythematosus (SLE) in IL-21R-competent *Yaa *mice, including hypergammaglobulinemia, auto-antibody production and reduced frequencies of marginal zone B cells [45]. Blockade of IL-21 with IL-21R-Fc fusion protein has resulted in fewer IgG glomerular deposits, circulating dsDNA auto-antibodies, total sera IgG1, IgG2a and lymphadenopathy in the lupus-prone MRL-Fas^lpr ^mouse [46]. Corresponding to therapeutic efficacy, administration of IL-21R-Fc fusion protein to BXSB.B6-Yaa+/J mice, another model of lupus, decreased lymphocyte activation and circulating IgG1 levels [47]. In our study, IL-21 induced B cell activation and proliferation could be reversed by anti-IL-21R antibody, which may indicate that IL-21 regulates B cell function via binding with IL-21R.

## Conclusions

Our results highlight that increased serum IL-21 levels in RA patients correlate with serum DAS28, anti-CCP antibody and the frequencies of Tfh-like cells. IL-21 supports B cell activation, proliferation and antibody secretion via the IL-21R pathway. All these data indicate IL-21 is involved in the pathogenesis of RA. Therefore, it opens avenues for the possibility that IL-21 might be targeted by therapeutic strategies for the clinical management of RA in the future.

## Abbreviations

anti-CCP: anti-cyclic citrullinated peptide; APC: allophycocyanin; CFSE: carboxyfluorescein diacetate succinimidyl ester; CRP: C-reactive protein; CS: corticosteroid; DAS28: 28-joint count disease activity score; DMARDs: disease-modifying antirheumatic drugs; ELISA: enzyme-linked immunosorbent assay; ESR: erythrocyte sedimentation; FCS: fetal calf serum; FITC: fluorescein isothiocyanate; GC: germinal centre; HC: healthy control; Ig: immunoglobulin; IL-21: interleukin-21; IL-21R: interleukin-21 receptor; NK: natural killer; NSAIDs: non-steroidal anti-inflammatory drugs; OA: osteoarthritis; PB: peripheral blood; PBMC: peripheral blood mononuclear cells; PBS: phosphate-buffered saline; PC: plasma cell; PE: phycoerythrin; PHA: phytohemagglutinin RA: rheumatoid arthritis; RF: rheumatoid factor; SEM: standard error of the mean; Tfh: T follicular helper.

## Competing interests

The authors declare that they have no competing interests.

## Authors' contributions

XL and LYS designed and directed the research. RL performed the experiments, analyzed and interpreted data and drafted the manuscript. QW, DLS and LYG collected the data. WJC, NC, HFC, and JYC provided critical input and edited the manuscript. All authors read and approved the final manuscript for publication.

## Supplementary Material

Additional file 1 **(a) The serum levels of IL-21 in Figure **2a. **(b) **The serum levels of IL-21 in Figure 3c. **(c) **The serum levels of IL-21 in Figure 3f. Click here for file

Additional file 2 **(a) The serum levels of IL-21 in Figure **4a. **(b) **The serum levels of IL-21 in Figure 4b. **(c) **The serum levels of IL-21 in Figure 4c. **(d) **The serum levels of IL-21 in Figure 4d. **(e) **The serum levels of IL-21 in Figure 5a. **(f) **The serum levels of IL-21 in Figure 5b. **(g) **The serum levels of IL-21 in Figure 5c. Click here for file
