# Supplementary figures and images for: A regulatory effect of IL-21 on T follicular helper-like cell and B cell in rheumatoid arthritis
Source: Arthritis Res Ther. 2012 Nov 23;14(6):R255. doi: 10.1186/ar4100 (PMC3674600; doi:10.1186/ar4100)

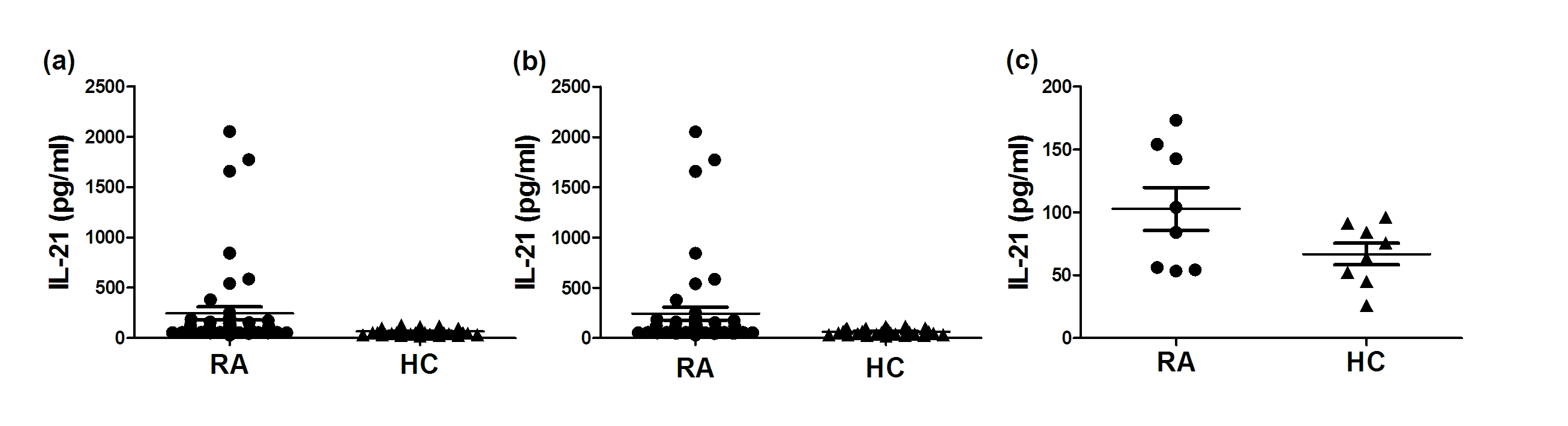

Supplement: Additional file 1 — (a) The serum levels of IL-21 in Figure 2a. (b) The serum levels of IL-21 in Figure 3c. (c) The serum levels of IL-21 in Figure 3f. [file ar4100-S1.TIFF]

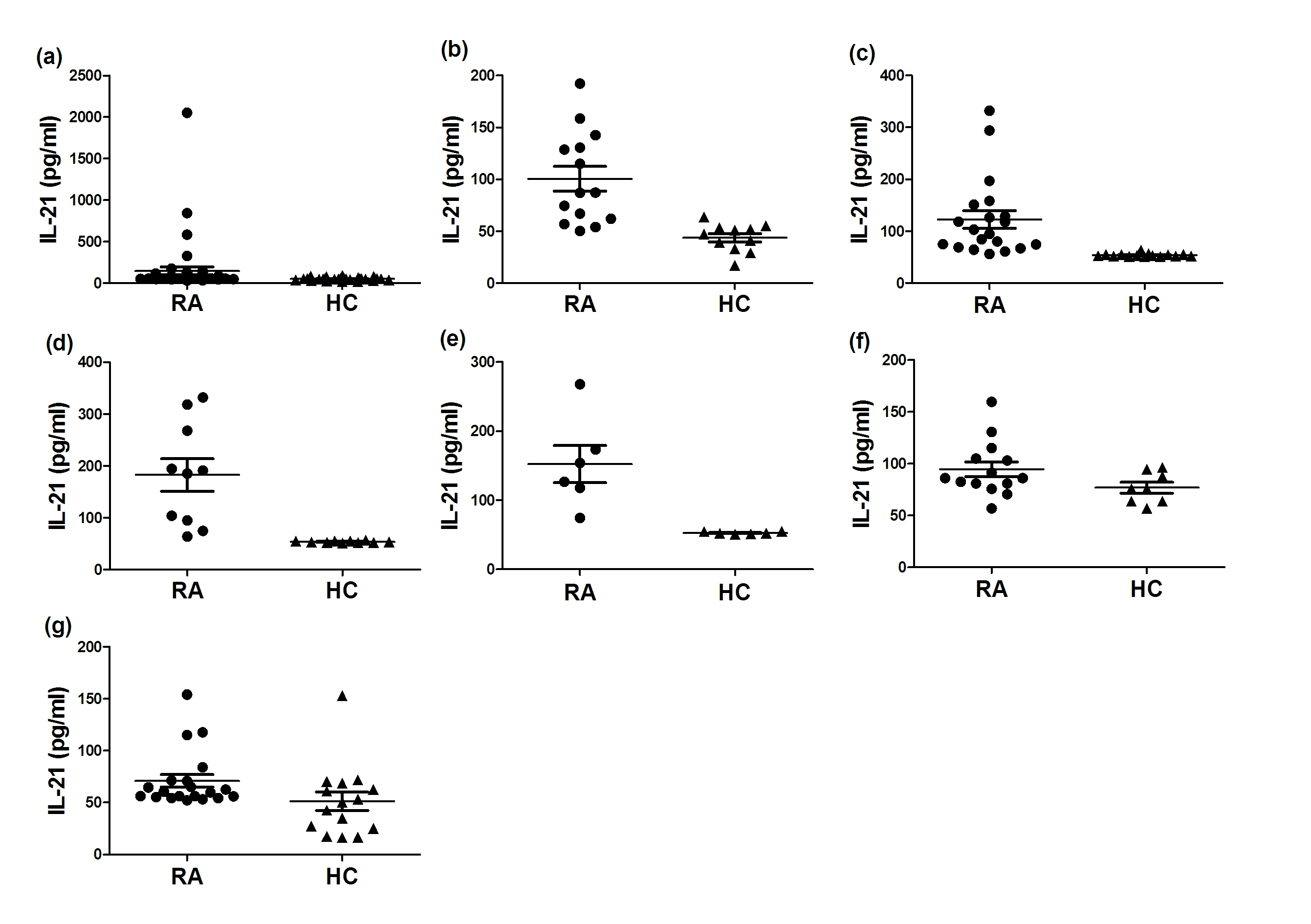

Supplement: Additional file 2 — (a) The serum levels of IL-21 in Figure 4a. (b) The serum levels of IL-21 in Figure 4b. (c) The serum levels of IL-21 in Figure 4c. (d) The serum levels of IL-21 in Figure 4d. (e) The serum levels of IL-21 in Figure 5a. (f) The serum levels of IL-21 in Figure 5b. (g) The serum levels of IL-21 in Figure 5c. [file ar4100-S2.TIFF]
